# KCNJ11: Genetic Polymorphisms and Risk of Diabetes Mellitus

**DOI:** 10.1155/2015/908152

**Published:** 2015-09-13

**Authors:** Polin Haghvirdizadeh, Zahurin Mohamed, Nor Azizan Abdullah, Pantea Haghvirdizadeh, Monir Sadat Haerian, Batoul Sadat Haerian

**Affiliations:** ^1^Pharmacogenomics Lab, Department of Pharmacology, Faculty of Medicine, University of Malaya, 50603 Kuala Lumpur, Malaysia; ^2^Department of Pharmacology, Faculty of Medicine, University of Malaya, 50603 Kuala Lumpur, Malaysia; ^3^Department of Biology, Faculty of Science, Azad University, Tehran, Iran; ^4^Shahid Beheshti University of Medical Sciences, P.O. Box 19395-4763, Tehran, Iran; ^5^Food and Drug Control Reference Labs Center (FDCRLC), Ministry of Health and Medical Education, Tehran 131456-8784, Iran

## Abstract

Diabetes mellitus (DM) is a major worldwide health problem and its prevalence has been rapidly increasing in the last century. It is caused by defects in insulin secretion or insulin action or both, leading to hyperglycemia. Of the various types of DM, type 2 occurs most frequently. Multiple genes and their interactions are involved in the insulin secretion pathway. Insulin secretion is mediated through the ATP-sensitive potassium (KATP) channel in pancreatic beta cells. This channel is a heteromeric protein, composed of four inward-rectifier potassium ion channel (Kir6.2) tetramers, which form the pore of the KATP channel, as well as sulfonylurea receptor 1 subunits surrounding the pore. Kir6.2 is encoded by the potassium inwardly rectifying channel, subfamily J, member 11 (KCNJ11) gene, a member of the potassium channel genes. Numerous studies have reported the involvement of single nucleotide polymorphisms of the KCNJ11 gene and their interactions in the susceptibility to DM. This review discusses the current evidence for the contribution of common KCNJ11 genetic variants to the development of DM. Future studies should concentrate on understanding the exact role played by these risk variants in the development of DM.

## 1. Introduction

Diabetes mellitus (DM) is a chronic disease characterized by high blood glucose levels caused by either insufficient insulin production by the pancreas or improper response of the body cells to insulin [1]. Approximately 366 million people were diagnosed with DM worldwide in 2011 and this is expected to increase to 552 million by 2030 [2]. This disease has early- and late-stage complications. Early complications include hyperglycemia, polyphagia, polydipsia, polyuria, and blurred vision, leading to complications manifested later such as vascular disease, heart disease, stroke, peripheral neuropathy, nephropathy, and predisposition to infection [3].

DM is classified into various types, of which type 2 (T2DM) occurs most frequently. Approximately 5%–10% of patients with DM are affected by type 1 (T1DM) and more than 90% by T2DM. T1DM (formerly insulin-dependent diabetes, or juvenile diabetes) results from the autoimmune destruction of the insulin-producing beta cells in the pancreas [4]. T2DM (formerly noninsulin-dependent DM) is a metabolic disorder due to hyperglycemia in the context of insulin resistance and relative lack of insulin. This is in contrast to T1DM, in which there is an absolute lack of insulin due to the breakdown of islet cells in the pancreas [5]. Gestational DM (GDM) affects 3%–10% of pregnancies in various populations. In this disease, insulin receptors do not function properly, which results in high blood glucose levels during pregnancy with subsequent negative effects, such as the baby being large for gestational age, being born stillborn, or dying shortly after birth or the mother having a miscarriage or problems with her eyes and kidneys [6, 7].

DM is a multifactorial disease caused by both genetic and environmental factors and their complex interaction. Environmental risk factors include obesity, physical inactivity, hypertension, abnormal cholesterol levels, age, and smoking [8]. Pathogenic and nonpathogenic mutations in genomic DNA can also contribute to the development of diabetes. Several single nucleotide polymorphisms (SNPs), which are a type of nonpathogenic mutation, have been reported to be associated with susceptibility to different types of DM. The aim of this review is to assess the possible contribution of SNPs of the gene known as the potassium inwardly rectifying channel, subfamily J, member 11 (KCNJ11), which forms a compartment of the ATP-sensitive potassium (KATP) channel, present in beta cells of the islets, in the susceptibility to different types of DM.

## 2. Role of Genetics in the Development of DM

People with a family history of T1DM and T2DM are six and three times more likely, respectively, to develop these diseases than are unrelated individuals [9]. Multiple genes are involved in DM. Those that have garnered the most attention are the ATP-binding cassette transporter subfamily C member 8 (ABCC8) gene; the KCNJ11 gene; and the peroxisome proliferator-activated receptor-gamma (PPARG) gene. Most of these genes are involved in insulin action/glucose metabolism, pancreatic beta cell function or other metabolic conditions (e.g., energy intake/expenditure, lipid metabolism) [10]. Mutations in genes such as ABCC8 and KCNJ11 can disrupt the potentiation activity of the KATP channel and have thus been associated with permanent neonatal DM [11]. The PPARG gene is implicated in adipogenesis and the development of insulin resistance. Deleterious mutations in this gene impair insulin resistance and can cause lack of response to insulin [12].

From recent genome-wide association studies, more than 60, 500, and 65 loci have been identified for susceptibility to T1DM, T2DM, and GDM, respectively [13]. SNPs are the most common type of genetic variation distributed within or outside a gene region in the human genome. The frequency of SNPs is less than 1% in the genome, and approximately 54% of these variants are not deleterious [14]. SNPs can modify the risk of occurrence of a disease, either alone or in linkage disequilibrium in one gene or in neighborhood genes. For instance, in several studies, the common Pro12Ala polymorphism in the PPARG gene, the Glu23Lys polymorphism in the KCNJ11 gene, or the Ser1369Ala polymorphism in the ABCC8 gene was confirmed to be associated with DM [15].

## 3. KCNJ11 Gene and Its Product

**Figure 1:**
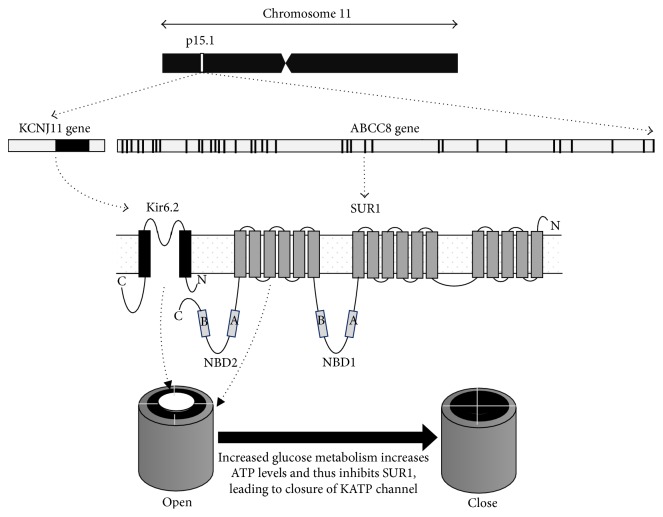
ABCC8 and KNJ11 genes and their encoded proteins and functions. The KCNJ11 and ABCC8 genes are next to each other on chromosome 11p15.1. KCNJ11 is a single exon (dark box) gene encoding the Kir6.2 protein, and ABCC8 has 35 exons (dark boxes) encoding the SUR1 protein; both are subunits of the ATP-sensitive potassium (KATP) channel. Metabolism of glucose can affect ATP levels and thereby the function of the KATP channel. ABCC8: ATP-binding cassette transporter subfamily C member 8; KCNJ11: potassium inwardly-rectifying channel, subfamily J, member 11; Kir6.2: inward-rectifier potassium ion channel; SUR1: sulfonylurea receptor 1; NBD1: nucleotide-binding domain 1; NBD2: nucleotide-binding domain 2; N: NH2 terminal of protein; C: COOH terminal of protein; A: Walker A motif; B: Walker B motif; cAMP: cyclic adenosine monophosphate; ATP: adenosine triphosphate.

The KCNJ11 gene, a member of the potassium channel gene family, is located at 11p15.1 and has no intron (Figure 1: 17,385,237–17,389,331). This gene encodes an inward-rectifier potassium ion channel (Kir6.2). The Kir6.2 protein, together with the high-affinity sulfonylurea receptor 1 (SUR1), forms the KATP channel. SUR1 is encoded by the ABCC8 gene located next to the KCNJ11 gene. The Kir6.2 protein is a 390-amino acid protein with two transmembrane domains (M1 and M2) and intracellular N- and C-terminals. Structurally, Kir6.2 tetramers form the pore and four high-affinity SUR1 subunits surround the pore of the KATP channel located at the plasma membrane of pancreatic beta cells. This channel modulates insulin production and secretion through glucose metabolism [16].

### 3.1. Role of Kir6.2 in Insulin Secretion

**Figure 2:**
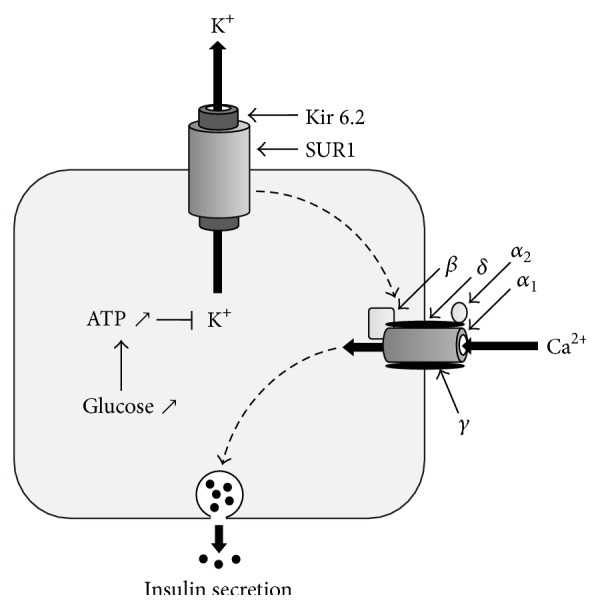
Mechanism of insulin secretion by the KATP channel in pancreatic beta cells. The Kir6.2 and SUR1 proteins in the KATP channel mediate insulin secretion. An increase in glucose levels stimulates the KATP channel to allow the entry of potassium ions. An increase in intracellular potassium ions depolarizes the cell membrane and induces calcium channels to increase intracellular free Ca^2+^ levels. The calcium ions trigger other components of the insulin secretion pathway to release granules at or near the plasma membrane. KATP: ATP-sensitive potassium channel; Kir6.2: inward-rectifier potassium ion channel; SUR1: sulfonylurea receptor 1; ATP: adenosine triphosphate; K^+^: potassium ion; Ca^2+^: calcium ion. The calcium channel is composed of *α*_1_, *α*_2_, *β*, *γ*, and *δ* subunits.

The Kir6.2 protein, coupled with the SUR1 protein in the KATP channel, mediates insulin secretion. This channel is involved in a wide range of physiological responses. Increased glucose induces higher potassium flow into the cell through the KATP channel. ADP in the presence of magnesium (Mg) converts to ATP; the ATP then closes the KATP channel by binding to Kir6.2, increasing the intracellular potassium ion concentration, which depolarizes the cell membrane and subsequently activates calcium ion (Ca^2+^) channel. Ca^2+^ is a ubiquitous intracellular second messenger that is critical for cellular functioning. These calcium channels influence the voltage-dependent potassium channels to repolarize the cell membrane, leading to closure of the voltage-dependent calcium channels. Increased intracellular free Ca^2+^ levels trigger other components of the insulin secretion pathway to release granules at or near the plasma membrane (Figure 2). Mutations in the* KCNJ11 *gene can cause DM because of the reduced ability of ATP to inhibit the activity of the KATP channel and the enhanced ability of MgATP to simultaneously stimulate the function of this channel. This is associated with defective insulin secretion, ultimately causing DM [17].

### 3.2. KCNJ11 Common Polymorphisms Involved in Diabetes

KCNJ11 has 219 SNPs, six of which have been receiving more attention for their association with diabetes. Among these six common SNPs, three are located in the coding regions and three in the noncoding regions (Table 1). These six SNPs include rs5219, rs5215, rs5210, rs5218, rs886288, and rs2285676.

#### 3.2.1. KCNJ11 rs5219

This locus is located in exon 1 of the KCNJ11 gene. Substitution of A to C (AAG→CAG) changes the amino acid from lysine to glutamine (Lys23Gln) at the NH2-terminal tail of Kir6.2. Lysine has a positively charged epsilon-amino group, whereas glutamine is uncharged under all biological conditions. Despite this amino acid substitution, theoretically, it does not make a remarkable change in the structure and function of the KCNJ11 protein [18]. Studies have shown, however, that the rs5219 variant may alter the charge of the ATP-binding region and decrease channel sensitivity to ATP. Twenty-four association studies and a recent meta-analysis showed a strong relationship between the rs5219 polymorphism and susceptibility to T2DM [19–43], whereas 21 studies did not confirm this finding [44–64]. This meta-analysis showed that the rs5219 polymorphism is a risk factor for developing T2DM in Caucasians and in some Asian populations. Populations from East Asia were more prone to this disease, where the A allele frequency in most patients was more common than in controls. Therefore, genetic background can affect susceptibility to T2DM [65].

The rs5219 polymorphism can affect the insulin secretion pathway. The A allele of this locus impairs this pathway by reducing ATP sensitivity of the KATP channel, hence resulting in overactivity of the channel and subsequent suppression of insulin secretion. This effect on insulin secretion is more significant in carriers of the AA genotype compared with carriers of the GA genotype [30]. Similar results were observed for fasting plasma glucose and postprandial plasma glucose levels in patients with T2DM. The A allele increased the fasting plasma glucose and postprandial plasma glucose levels in these patients, whereas GA carriers had higher 2 h postprandial plasma glucose levels than did GG carriers with T2DM [32, 38]. This allele was also associated with reduction in serum insulin levels in a postoral glucose tolerance test [39]. In contrast to one study from Scandinavia on GDM risk [37], the remaining studies did not report any association between this locus and T1DM and GDM [66–68].

Hypertension is a main complication of T2DM. The rs5219 polymorphism plays a strong role in HbA1c and blood pressure levels in this disease. The A allele carriers of rs5219 had higher HbA1c levels and blood pressure than did the G allele carriers [33, 35, 36, 40, 41]. In T2DM, a relationship has been suggested between the A allele and increased hepatitis insulin sensitivity [32]. Pharmacogenomics studies demonstrated that A allele carriers of the rs5219 polymorphism who have T2DM have better therapeutic response to gliclazide than do G allele carriers. In the A allele group, HbA1c was also reduced more in patients taking glimepiride and glibenclamide than it was in patients taking gliclazide treatment [40]. The ring-fused pyrrole moiety in these two drugs binds to the A allele, underling the inhibitory potency of these drugs on KATP channels [41]. The rs5219 polymorphism also plays a role in determining the efficacy of repaglinide [33, 39]. Carriers of the C allele were also found to have a reduced response to sulfonylurea therapy [42, 43].

#### 3.2.2. KCNJ11 rs5215

The rs5215 polymorphism is located in exon 1 of the KCNJ11 gene. It is a nonsynonymous variant caused by a substitution of G to A (GTC→ATC), which changes the amino acid from valine to isoleucine at residue 250. Valine is hydrophobic, whereas isoleucine is one of three amino acids having branched hydrocarbon side chains. Isoleucine is usually interchangeable with leucine and occasionally with valine in proteins. Of 13 studies on DM, 3 showed strong associations between this variant and T2DM [27, 30, 35], whereas the remaining studies showed no association with T2DM, T1DM, or GDM [36, 49–51, 69–74]. In another study, the rs5215 polymorphism was associated with blood pressure among subjects with T2DM [36].

#### 3.2.3. KCNJ11 rs5210

The rs5210 polymorphism is located at a highly conserved 3′ untranslated region (UTR) of the KCNJ11 gene. Of four reports relevant to susceptibility to T2DM, two identified a plausible role in development of this disease, whereas the other studies did not confirm this relationship [30, 36, 72, 75]. A study found that this variant improves the clinical efficacy of gliclazide in patients with T2DM [76]. This locus is a target of miR-1910; however, the mechanism of action of this miRNA in the development of DM is unknown. MiRNAs encompass 17 to 25 nucleotides, which posttranscriptionally regulate the expression of thousands of genes in a broad range of organisms in both normal physiological and disease contexts. Appropriate secretion of insulin from pancreatic beta cells is a vital factor in blood glucose homeostasis, and miRNAs have been identified as being involved in the regulation of insulin exocytosis. MiRNAs control insulin synthesis and release it in beta cells. The G allele is a potential target for miR-1910, whereas the A allele abolishes binding of this miRNA to this region [77, 78]. Further studies may reveal the role of miR-1910 in DM.

#### 3.2.4. KCNJ11 rs5218

The rs5218 polymorphism is located in the 3′-UTR of the KCNJ11 gene. It is a synonymous variant with a substitution of G to A (GCC→GCT), which encodes, for alanine at residue 103, a hydrophobic and ambivalent amino acid. There is only one report of this locus in DM, which showed no association with T2DM risk [36].

#### 3.2.5. KCNJ11 rs886288 and rs2285676

The rs886288 polymorphism is located in the 5′ flank near the gene, whereas the rs2285676 polymorphism is located at the 3′-UTR region. Two studies revealed an association of the rs886288 and rs2285676 polymorphisms with T2DM [30, 36].

### 3.3. Interaction of the KCNJ11 Gene with Other Genes

**Figure 3:**
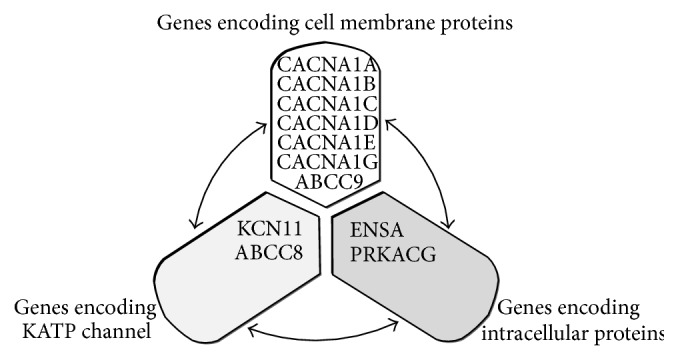
Cross-talk between the KCNJ11 gene and the other genes involved in the regulation of insulin secretion in pancreatic beta cells. KCNJ11 interacts with many genes, of which 10 are most prominent. KCNJ11 interacts with ABCC8 to produce the KATP channel, which transfers potassium ions across the beta cells. The KCNJ11 and ABCC8 genes interact with three groups of gene products at the cell membrane (white) and the intracellular (dark gray) levels. KATP: ATP-sensitive potassium channel; CACNA1A: calcium channel, voltage-dependent, P/Q type, alpha 1A subunit; CACNA1B: calcium channel, voltage-dependent, N type, alpha 1B subunit; CACNA1C: calcium channel, voltage-dependent, L type, alpha 1C subunit; CACNA1D; calcium channel, voltage-dependent, L type, alpha 1D subunit; CACNA1E: calcium channel, voltage-dependent, R type, alpha 1E subunit; CACNA1G: calcium channel, voltage-dependent, T type, alpha 1G subunit; ABCC9: ATP-binding cassette transporter subfamily C member 9; KCNJ11: potassium inwardly rectifying channel, subfamily J, member 11; ABCC8: ATP-binding cassette transporter subfamily C member 8; ENSA: endosulfine alpha; PRKACG; protein kinase catalytic subunit G; RAPGEF4: rap guanine nucleotide exchange factor 4; FOXA2: forkhead box A2.

Insulin secretion from pancreatic beta cells can be modulated by a complex cluster of proteins encoded by related genes, including KCNJ11, ABCC8, voltage-sensitive calcium channels (VSCCs), ABCC9, protein kinase catalytic subunit G (PRKACG), rap guanine nucleotide exchange factor 4 (RAPGEF4), forkhead box A2 (FOXA2), and endosulfine alpha (ENSA). These proteins act at the cell membrane or intracellular level (Figure 3).

#### 3.3.1. Interactions at the Cell Membrane Level

KCNJ11 and ABCC8 genes encode Kir6.2 and Sur1, respectively, in pancreatic beta cells. Both proteins form compartments in the KATP channels, which allow potassium to flow into the cell rather than out of it, as mediated by G proteins [17]. The KATP channel interacts with different types of VSCCs, including L (long-lasting), N (neural), P/Q (purkinje), R (residual), and T (transient). Calcium channels are generally composed of four subunits: *α*_1_, *α*_2_-*δ*, *β*, and *γ*. The function of the calcium channel is controlled by the pore-forming *α*_1_ subunit, which blocks the entry of calcium ions into the excitable cells, and by the auxiliary subunits, which modulate trafficking and the biophysical properties of the *α*_1_ subunit. The *α*_1_ subunit isoforms include A, B, C, D, E, and G, encoded by CACNA1A, CACNA1B, CACNA1C, CACNA1D, CACNA1E, and CACNA1G genes, respectively. The A to E forms of the *α*_1_ subunit produce various types of calcium channels, including P/Q, N, L, L, R, and T, respectively. The L, N, P/Q, and R types of these channels belong to the high-voltage activated (HVA) group and the T type belongs to the low-voltage activated (LVA) group. Both HVA and LVA groups are involved in calcium-dependent processes such as neurotransmitter or hormone release, muscle contraction, cell motility, gene expression, cell division, and cell death [78, 79]. Finally, Kir2 and ABCC9 can form another type of KATP channel in cardiac, skeletal, vascular, and nonvascular smooth muscle. The structure of the ABCC9 protein suggests a role as a drug-binding, channel-modulating subunit of the extrapancreatic KATP channels [80].

#### 3.3.2. Interactions at the Intracellular Level

The KATP channels interact with the PRKACG protein encoded by the PRKACG gene. This protein is the gamma catalytic subunit of protein kinase, which is involved in exocytosis through different pathways such as calcium- and hormone-mediated signaling. This protein also activates cellular processes such as intracellular protein kinase A [81]. Kir6.2 interacts with RAPGEF4, FOXA2, and ENSA proteins, encoded by RAPGEF4, FOXA2, ENSA, and ABCC9 genes, respectively. RAPGEF4 is an exchange protein that can be activated by cAMP. FOXA2 functions as a transcription activator for genes such as alpha-fetoprotein, albumin, and tyrosine aminotransferase. ENSA is an endogenous ligand for SUR1, which stimulates insulin secretion [82–84]. Defects in the KCNJ11 gene may also lead to autosomal-dominant T2DM, transient neonatal DM type 3, and permanent neonatal DM [85].

## 4. Concluding Remarks

DM is one of the most common diseases globally, with high social and economic burdens. Kir6.2 plays a potential role in the function of the KATP channel. Some active mutations in this gene can disrupt Kir6.2 activity and consequently reduce the potential of the KATP channel, leading to DM. It is evident from the literature that several variants of the KCNJ11 gene are associated with different types of DM. This raises the question of which polymorphisms of the KCNJ11 gene and their combinations play more prominent roles in the development of DM.

Most previous studies have focused on six common polymorphisms in DM: rs5210, rs5215, rs5218, rs5219, rs886288, and rs2285676. Of these six loci, rs5219, rs5215, and rs5210 have been given most attention. No evidence yet exists in the literature for the involvement of other SNPs of the KCNJ11 gene. The rs5219 A allele plays an important role in insulin secretion through reduction of ATP sensitivity of the KATP channel and suppression of insulin secretion. However, the mechanism of action of this locus in the insulin secretion pathway is still not completely understood. The rs5210 G allele acts as a potential target for miR-1910, which is implicated in T2DM; however, the mechanism of action of this miRNA in the development of DM is unknown. MiRNAs control insulin synthesis and release from beta cells. Future studies are suggested to reveal the use of miR-1910 as a potential biomarker in the diagnosis of diabetes and its plausible application for treatment of DM.

Regulation of insulin release is mediated by KCNJ11 in concert with different genes such as ABCC8, ABCC9, and CACNA1A-G. Diminished coexpression of these genes may increase the risk of DM. Nevertheless, the exact functional relationship of the network of these genes in the regulation of insulin release remains to be determined. Future studies are suggested to discover the exact role of KCNj11 gene variants and their interaction with other genes in DM for the possible development of suitable therapies and the diagnosis of this common disease.

## Figures and Tables

**Table 1:** Characteristics of KCNJ11 gene variants in association with diabetes mellitus.

Number	SNP	Location		MAF	Allele	Amino acid	Diabetes	Association	References
		Chromosome	Gene						
1	rs2285676	17386478	3′ UTR	0.46	T>C	—	T2DM	Yes	[30]

2	rs5210	17386704	3′ UTR	0.46	G>A	—	T2DM	Yes	[30, 36, 76]
								No	[72, 74]

3	rs5215	17387083	Exon	0.28	G>A	Val250Ile	T2DM	Yes	[27, 30, 35, 36]
								No	[49, 50, 69–72]
							T1DM	No	[73, 74]
							GDM	No	[68]

4	rs5218	17387522	Exon	0.27	C>T	Ala103Ala	T2DM	No	[36]

5	rs5219	17388025	Exon	0.27	G>A	Lys23Gln	T1DM	No	[63, 64]
							T2DM	Yes	[19–43]
								No	[44–64]
							GDM	Yes	[38]
								No	[66–68]

6	rs886288	17389616	5′ near gene	0.46	T>C	—	T2DM	Yes	[36]

MAF: minor allele frequency; T1DM: type 1 diabetes mellitus; T2DM: type 2 diabetes mellitus; GDM: gestational diabetes mellitus; SNP: single nucleotide polymorphism; UTR: untranslated region.
